# “It's Not Just in My Head, and It's Not Just Irrelevant”: Autistic Negotiations of Menopausal Transitions

**DOI:** 10.1007/s10803-021-05010-y

**Published:** 2021-04-22

**Authors:** Marianna Karavidas, Richard O. de Visser

**Affiliations:** 1 School of Psychology, University of Sussex, Brighton, UK; 2 Present Address: Department of Primary Care & Public Health, Brighton & Sussex Medical School, Falmer, BN1 9PH UK

**Keywords:** Autism, Menopause, Wellbeing, Qualitative, Neurodiversity, Critical realism

## Abstract

Physical and psychological changes during menopause can be especially tumultuous for autistic people: difficulties with sensory sensitivity and daily functioning may be exacerbated. Through individual interviews, we examined the language used by seven peri- or post-menopausal autistic people to construct their experiences, and to consider the implications for their wellbeing and identities. Our analysis, which utilised thematic decomposition, yielded three discursive themes. The theme “Uncertainty about Changes” addressed how limited awareness and understanding of menopause combined with difficulties recognising internal states. However, with “Growing Self-Awareness and Self-Care”, some participants made conscious efforts to resist negative societal constructions of both autism and menopause. The theme “Navigating Support Options” addressed the interpersonal and systemic barriers participants faced when seeking support. There is a need for accessible information for autistic people experiencing menopause, and greater professional awareness.

The intersection of autism and menopause has—until recently—received little attention. Autism is a neurodevelopmental condition associated with differences in social communication and understanding, sensory sensitivity and preferences for routines and familiarity (American Psychological Association [APA], 2013). It is considered to be underdiagnosed among those assigned female at birth (Lai & Baron-Cohen, 2015), however an increasing number are receiving diagnoses as adults (Chester, 2019), an explanation for their experiences and their sense of self that may have been unavailable to them during childhood adolescence, and earlier adulthood. Autism and menopause both have implications for people’s experiences and sense of self. Late recognition of autism may mean that people have to adapt to this new component of their identity near the time when they also need to adapt to menopausal changes.

Sexual embodiment is a term used to emphasise that women’s experiences of their bodies are located within the cultural context in which they live (Jackson & Scott, 2007). Material (physical), discursive (cultural and social) and intrapsychic (psychological) factors intersect to influence all subjective experiences, including transition points such as menopause (Bhaskar, 2010). At a material level, menopause is defined as the permanent cessation of menstruation: the median age ranges from 49 in Latin America to 54 in Europe (Palacios et al., 2010), and the mean age ranges from 47 in Latin America and the Middle East to 51 in Australia and Europe (Schoenaker et al., 2014). Perimenopause is the period of several years leading up to this point, characterised by gradual physical and endocrine changes that affect neurotransmitter systems (Santoro, 2016). Risks of developing clinical depression, autoimmune diseases and other health issues increase around the time of both menopause and menarche (Hoyt & Falconi, 2015; Pinkerton & Stovall, 2010).

Cultural discourses are suggested to be the framework through which individuals make sense of material and intrapsychic menopausal changes, with implications for the extent of the distress and discomfort they experience (Tolman et al., 2014). In Western cultures, the menopausal body has tended to be examined through a biomedical lens; changes are viewed as undesirable “symptoms” (Ussher, 2008). However, such constructions are by no means universal. Discursive differences appear to have important material effects: women have reported more frequent hot flushes and subsequent embarrassment and more menopause-related health concerns in cultures that position menopause as a negative medical issue, relative to those where this life stage is viewed in a more neutral or positive light (Sayakhot et al., 2012).

There is a paucity of research into the topic of autistic menopause specifically. However, there is evidence that material and intrapsychic aspects of reproductive transition points may be especially tumultuous for autistic people (e.g., Hamilton et al., 2011). Premenstrual syndrome (symptoms such as low mood and headaches prior to menstruation) may be more common among women with an autism diagnosis than those without (Obaydi & Puri, 2008), and domains that are specific to autism such as communication difficulties and sensory sensitivity appear to undergo cyclical amplifications (Steward et al., 2018). Moseley, Druce and Turner-Cobb’s (2020a, 2020b) recent papers were the first to investigate autistic menopause. Notably, participants described uncomfortable increases in autistic traits such as sensory sensitivity, and social and emotional regulation difficulties.

Like menopause, autism has traditionally been viewed through an abject biomedical lens of “persistent deficits in social communication” (APA, 2013). Adult autism diagnoses are often only reached in the context of longstanding mental and physical health issues or a history of abusive experiences (Bargiela et al., 2016 Roberts & Koenen, 2015; Cashin et al, 2018). Indeed, a history of mental and physical health conditions and abusive experiences in early life are associated with greater menopausal discomfort (Pérez-López et al., 2014; Thurston et al., 2008). However, many late-diagnosed autistic women describe personal growth as a result of overcoming obstacles throughout their lives: diagnosis had positive implications for wellbeing (Webster & Garvis, 2017). Moseley et al. (2020b) asked questions such as “Are there things that you think might make menopause especially difficult for autistic women?” which enquired about negative, or clinically concerning changes that they faced. However, Moseley et al. (2020a) also identified a subtheme of “silver linings”: despite being the source of “dark clouds” that affected sexual relationships, appearance, and self-esteem, menopause also provided benefits such as cessation of periods, adoption of healthier lifestyles, and reflection on priorities in inter-personal interactions.

Internalisation of damning biomedical discourses has been suggested to lead to women viewing their changing bodies as objects of secrecy and shame: when talking about menopause, women often use euphemisms (Hunter & O’Dea, 1997). Women with learning disabilities have described embarrassment about menopause which prevented them from disclosing uncomfortable changes to helpers (McCarthy & Millard, 2003). Autism is associated with impaired comprehension of social norms, however autistic females have a tendency to camouflage autistic traits in order to conform socially (Perry, 2019). Autistic women have been found to express apprehension about discussing menopause with peers, and many avoid doing so (Moseley et al., 2020b). However, experiential knowledge and solidarity shared between female peers in the general population is often a valued source of support during menopause (Kenen et al., 2007). Autistic people often have small social networks (Mazurek, 2014): it is possible that their differences in social milieux and understanding may influence their internalisation of cultural discourses and their subsequent treatment decisions. This is an important consideration given that autistic people have reported significant difficulties with healthcare services (Lum et al., 2014) and are at an increased risk of premature all-cause mortality and morbidity (Hirvikoski et al., 2016).

The present study aimed to understand autistic people’s own understandings of the positive and negative aspects of menopausal change. The research questions were: How do autistic people discursively construct their experiences of menopausal change? What are the implications of these constructions for wellbeing, identity and accessing support?

## Methods

### Participants

Participants were eligible if they had reached menopause or perimenopause, were assigned female at birth, did not have intellectual disabilities, and were based in the UK. There were no exclusion criteria regarding gender identity. Participants were eligible if they had a professional diagnosis or self-identified as autistic. This approach is considered good practice within autism research (Happé & Charlton, 2012) as it avoids prioritising testing procedures that may have a gender bias (Lai et al., 2015), and because it validates self-identified positions. Having said that, six of the seven participants had a professional diagnosis.

Table 1 displays participant pseudonyms and characteristics. Age at interview ranged from 39 to 63 years (mean = 49.4), age of recognition of autism ranged from 37 to 60 years (mean = 46.8), and the onset of menopausal changes ranged from 34 to 56 years (mean = 41.7). In the UK, menopause is usually identified through self-report of menstruation in the past 12 months (Chaplin, 2016): four participants identified as post-menopausal, three identified as perimenopausal.

**Table 1:** Participant pseudonyms, pronouns and characteristics

Pseudonym	Age	Pronouns	Self-reported menopausal status
Ash	47	They/ them/ their	Perimenopausal (change pinpointed at age 34)
Dora	52	She/ her/ hers	Post-menopausal (change pinpointed at age 40)
Ella	45	She/ her/ hers	Perimenopausal (change pinpointed at age 42)
Grace	63	She/ her/ hers	Post-menopausal (change pinpointed at age 47)
Isla	57	She/ her/ hers	Post-menopausal (change pinpointed at age 56)
Nina	39	She/ her/ hers	Post-menopausal (change pinpointed at age 36)
Olivia	43	She/ her/ hers	Peri-menopausal (change pinpointed at age 37)

Critical realist approaches do not offer specific sample-size recommendations. The aim should be *analytical generalisability*, i.e., the identification of underlying meanings and mechanisms rather than *statistical generalizability*; attempting to ascertain frequencies of a phenomenon or aiming for a comprehensive description of all aspects (Yin, 1989). Nevertheless, Malterud et al. (2016) have advised researchers to make initial decisions about an appropriate sample size, and to consider this on the basis of emerging findings.

### Procedure

The study was granted ethical approval by the authors’ Institutional Review Board. Potential participants were recruited via an advertisement on social media. Those who responded to the advertisement received a participant information sheet that outlined the topics that would be covered, right to withdraw, and data handling procedures. Informed consent was established through an electronic consent form.

The interview schedule was devised by the authors to explore the impact of autism diagnosis and menopause onset, experiences of bodily changes, and instances of seeking support or treatment. The semi-structured format ensured that core questions were asked of all participants while allowing flexibility to explore issues that were important to interviewees. Follow-up questions were worded as open-ended invitations for elaboration: e.g., “Can you give me an example?” (Magnusson & Marecek, 2015). Care was taken to avoid unnecessarily vague or figurative language, as this can confuse autistic people (Nicolaides et al., 2019). Additionally, effort was made to use neutral terminology, i.e., “menopausal changes” rather than “symptoms” in order to avoid priming a medical discourse.

Those willing to take part could choose to be interviewed in person or via Skype. Although it has been suggested that video interviewing may be less effective for building rapport than face-to-face interviews (Weller, 2017), this approach may be more accessible for autistic people (Zolyomi et al., 2019). Indeed, all opted for Skype interviews. Audio recordings were transcribed verbatim. Within 3 weeks of the interview, and prior to analysis, participants were emailed their transcripts and given the opportunity to “member check” the content (Creswell & Miller, 2010). Three participants made minor amendments.

### Analysis

We used a material-discursive-intrapsychic approach embedded within a critical realist epistemology (Tolman et al., 2014). This has been used extensively and effectively to explore women’s embodied experiences in various contexts (Ussher, 2008; Ussher et al., 2019). Critical realism is a perspective that recognises the materiality (or lived experience) of the body, but conceptualises this materiality as being mediated by discourse, culture and psychological factors (Bhaskar, 2010). This approach was chosen because the focus on language facilitated closer examination of the interactions between the material body, psychological processes and culture than can be achieved through approaches such as interpretative phenomenological analysis or narrative methods, which are primarily concerned with subjective experience (Willig, 2007).

Some proponents of the neurodiversity movement consider distress and disability to be entirely socially constructed and may argue that critical realist approaches problematically locate the cause of disability within the autistic person (MacDonald, 2019). However, a strict constructivist stance would deny the existence (or materiality) of autistic traits, including positive aspects such as the capacity for intense fascination and enjoyment of sensory stimuli (Woods et al., 2018). Furthermore, although cultural discourses have been suggested to influence the extent of menopausal discomfort, this discomfort is experienced within the material body (Stephens et al., 2004). The experience of menopause is neither entirely biomedical nor entirely socially constructed. A critical realist epistemology would acknowledge social aspects (i.e., influence of the medical model, ableism and stigma (De Jaegher, 2013)), meanwhile ensuring that the potentially tumultuous material impacts of menopause for the body and mind are not lost amongst more abstract discussion of discourse (Bhaskar, 2010).

Analysis was informed by thematic decomposition approaches, which attempt to identify themes in language that reflect subjective positions taken up by participants (Stenner, 1993). The process of coding involved re-reading transcripts and assigning each line an initial code on the basis of its content. Each transcript was coded and analysed independently of other transcripts, and without consideration of the themes from other transcripts. Subsequently, the codes from the entire corpus of interviews were entered into spreadsheets and were consolidated by looking for higher-level constructs represented by clusters of similar comments (Yin, 1989). The two authors independently coded one transcript, and then compared notes to agree on a consistent and comprehensive approach to analysis. For the remaining interviews, the first author conducted the initial analysis and discussed the spreadsheet of themes with the second author, who verified them against the transcripts.

The planning and execution of the study were informed by various recommendations for quality and validity in qualitative research (Malterud, 2001; Shaw, 2010; Yardley, 2000). *Reflexivity* (Malterud, 2001) and *sensitivity to context* (Yardley, 2000) were addressed by identifying the personal, micro-social, and macro-social influences on interviewees’ experiences, and by reflexively clarifying our own relationships to the issues. Critical realist approaches emphasise the importance of self-reflexivity: personal experiences and cultural discourse influence researchers as well as participants (Willis, 2019). Given that a ‘double empathy’ problem has foreclosed mutual understanding between autistic subjects and (often non-autistic) researchers (Milton, 2012), transparency about researchers’ relationship to the autism spectrum is important (Howard et al., 2019). The interviewer and first author was a 22-year-old cisgender female student with no experience of menopause who received an autism diagnosis in adulthood. The second author was a 48-year-old neurotypical cisgender man. Throughout the processes of data collection and analysis, we reflexively engaged in iterative processes of “setting aside” and “engaging with” our subjectivities by reflecting on our “insider” and “outsider” perspectives, and how these may have influenced the analytic process (Shaw, 2010). During the interviews, nonverbal interactions that would not be evident from the audio recording were noted. Notes were taken throughout the research process as an aid to reflection on personal thoughts and wider reading. Both were referred to during analysis as a means of ensuring that interpretations were rooted in the data, and not unduly influenced by topics that were personally significant to the researcher. In relation to *interpretation and analysis* (Malterud, 2001), *transparency and coherence* (Yardley, 2000) were demonstrated by clearly explaining how themes were generated, and by showing how themes were grounded in the data. *Commitment and rigor* (Yardley, 2000) were promoted by the authors independently coding transcripts before conferring to ensure a rigorous and consistent process. Reliability was ensured by regular communication between the authors to resolve differences of perspectives on emerging themes. *Impact and importance* (Yardley, 2000) and *transferability* (Malterud, 2001) were demonstrated by showing how the results could inform better healthcare interactions for autistic people during menopause.

## Results

Thematic decomposition resulted in the identification of three major discursive themes—*Uncertainty about Changes*, *Growing Self-Awareness and Self-Care,* and *Navigating Support Options*—each consisting of sub-themes. Each theme is described below and illustrated with quotes from the interview transcripts. Although these are presented as separate entities, the considerable overlaps will be explored further in the discussion.

### Uncertainty About Changes

A discourse of feeling uncertain complicated participants’ recognition of, and adjustment to, menopausal changes. This difficulty was attributed to insufficient prior information; societal discourses about menopause which were felt to be misleading; the timing of the autism diagnosis in relation to menopause onset; and long-standing difficulties interpreting bodily sensations within themselves which they retrospectively attributed to autistic traits.

#### Getting Ill?

Dora described uncomfortable physical and emotional changes: “When I turned 40, it was like everything started to go wrong” and was prescribed antidepressants. She viscerally described the arduousness of the experience: “It just felt like I was walking in mud for years… It was like a bad dream”. “Brain-fog”, a slang term for a symptom of temporary loss in mental clarity was contrasted with prior clarity of focus which caused Dora to question: “What is wrong with me? I'm an incompetent human”. Lack of explanation for changes led participants to adopt a discourse of self-criticism: the cause of these participants’ discomfort, rather harshly, was located within personal weaknesses. In hindsight, Dora attributed this disruption to a “very strong perimenopausal brain activity”. Although this description of menopausal neuroscience appeared to be situated within a biomedical discourse, she made a distinction between discomfort caused by menopause and the status of being mentally unwell:

I don't see it as me being depressed. I'm not a depressed person, but clearly the signs were that something was not at the right level. I wasn't functioning at all.

She retrospectively opposed the medicalisation of her situation by doctors by questioning the assumption that the menopausal body is abject: doctors “tell women they are really sick, when actually what we are going through is a really difficult transition time”. She recalled a particularly dismissive response from a medical professional who told her that discomfort during menopause was “all in her mind”.

Ash, who was already receiving treatment for clinical depression, initially attributed menopausal changes to “a medication problem”. Internalising this biomedical discourse delayed their recognition of menopause. However, for Ella: “the first thing that was kind of a flag for me was that I started getting worse anxiety symptoms”. For her, a biomedical discourse relating to her existing anxiety diagnosis and medication was positioned as material marker which helped her identify this change.

Ash acknowledged the discursive tendency for menopause to be kept hidden: “nobody tells you this shit … as a woman”. Six of the seven participants described significant discomfort during menopause, a lack of prior awareness was described as making menopause additionally confusing for all participants:

If I'd known about it before-hand I'd be able to say, ‘Oh yes, maybe the migraines were menopausal, maybe this was menopausal, maybe this was menopausal’, but it’s all passed me by because I didn't know about the menopause. (Isla)

Olivia reflected on the autistic tendency to experience awareness of bodily states as “either the hypo- or the hyper- … We never fall in the middle”. Isla considered that she may have simply overlooked menopausal symptoms. However, Grace described being unusually observant: “I noticed a lot of very subtle things that most people wouldn't”, such as changes in body hair.

#### Autistic Traits Come to the Surface

Heightened sensory sensitivity is not a “classic” menopausal symptom, but it is an autistic trait that has been reported to increase during menopause (Moseley et al., 2020b). Olivia described confusion resulting from temperature regulation changes:

My body has all kinds of strange sensory reactions and um ... I don't read it particularly well at the best of times ... It was just another thing that annoyed me about my body.

Likewise, Nina felt “out of sorts and horrible… really confused” and doubted herself: “You start to think, ‘Am I putting this on just because I want to get an [autism] diagnosis, or am I exaggerating?’”. Nina’s menopause was medically induced, and unlike Olivia, she was aware that she was undergoing menopause. However, neither had learnt that they were autistic at this point. Lack of awareness was positioned as adding to their distress. In hindsight, recognition of the combination of autism and menopause was positioned as a helpful explanation: “There is something in those hormonal shifts that brings [autism] to the surface … that makes it more prominent” (Olivia). Ash described a visceral intrapsychic process akin to a flashback during perimenopause:

It felt like I was a kid again and lots of the memories from when I felt uncomfortable as a child were flooding back; it was strange. I started to have vivid dreams from stuff years and years ago about stuff that had bothered me. In the light of autism, which I now know about, it made sense.

Grace recalled becoming intensely tearful during perimenopause, which coincided with difficult personal circumstances:

I was with a guy who I shouldn't have been with, but I was often very, very upset about the way he was and the things he said and so on, so I was just crying, and you know, just overwhelmed with emotion a lot of the time.

However, in post-menopause: “Sometimes I want to cry, and I can't. I think, ‘Oh, I should cry about that. And it would be good if I did. I'd feel better’. But it doesn't happen”. She humorously attributed this shift to a mysterious disappearance of hormones, “I wonder whether you know, I joke I say, “Oh, I have no hormones, so I have no tears”. However, she also considered an intrapsychic explanation for this change:

I worry sometimes, ‘What have I become?’… what's the word? Have I become dissociated? I can’t cry because I'm dissociated. I don't know. But I don't cry much now, hardly ever.

Dissociation is a psychological self-preservation response to severe stress (Knipe, 2018). Elsewhere in her interview, Grace explained that she had been “given an appointment to be assessed for PTSD”. Her autism assessment process involved reflecting on (and medicalising) negative experiences:

It’s all about the negatives, because that's how the diagnostic criteria. That's what they're looking for. They're looking for difficulties in, or impairments in. So, you're, you're filtering back over your life and you're going: ‘Oh, bloody hell! Yeah, it's amazing I’m still sane!’, if I am sane.

For many interviewees, distress was positioned as proportionate to real-life circumstances but was mediated by hormonal changes and reconceptualised in the light of a diagnosis of autism:

It seems like we ignored a lot of our traits, or just pushed through and didn't really recognize them. We appear more autistic to ourselves after we've received our diagnosis just because we have got the right perspective this time. But I do feel like - I do feel like it is ... worse than before. I just feel like the mood swings at everything I ... have always ... emotional regulation has always been an issue ... just feels like everything is amplified now. (Olivia)

#### Menstrual Changes: A Material Indicator of Menopause?

The materiality of menstrual changes was positioned as an objective indicator of menopause. For example, Ella noted: “You know what your cycle is normally and occasionally you have an unusual one, but this was definitely like every cycle was getting shorter, getting heavier, more painful”. However, Isla was unable to reconcile her experiences with the accounts available to her, meaning that menopause was misrecognised:

No one else has told me that they don't have these symptoms … The only symptom that I had was stop - my periods stopping … And the first period I missed I thought, ‘Oh, am I pregnant’ … and I thought ‘No, I can't be!’

Grace discursively positioned this change as unsettling due to the autistic tendency to dislike disruption to usual routine:

It’s very disturbing and, you know, now I understand why actually. You think you know, ‘Four weeks, four weeks, four weeks’, and then you're like ‘What! No, that's not right!”

However, post-menopausal participants such as Isla noted that the eventual cessation of periods was a relief: “One less thing to worry about” (Isla).

#### Uncontrollable and Unpredictable Menopause

The unpredictability of menopause led many participants to position menopause discursively as separate to themselves. Olivia described her perimenopausal body as a force beyond her control:

My body does its own thing and I've got to go along with whatever ride it's taking me on that day kind of thing. But no, I don't feel like I have any control over it at all.

Although she was not aware of disruptive menopausal changes, Isla highlighted that “next week I might want to contact you and say … ‘ugh … I've had an awful week … menopause has hit me on the head’”. Dora considered menopause to be an acceptable natural stage: “There is a real rhythm to life… I do not despise it”, but hastened to add, “I hope it stays like this”. Nina described a sense of relief that her medically-induced menopause was planned rather than “creeping up without me wanting it”. Dora highlighted the importance of preparing autistic people for life transitions: “With autistic girls you need to plan for puberty way before puberty. It's the same with ageing and menopause”. These quotes suggest that menopause would be more tolerable if participants knew what to expect.

### Growing Self-Awareness and Self-Care

The arrival of menopausal symptoms in addition to other age-related factors was positioned as the catalyst for becoming more aware of personal wellbeing needs.

#### Pushing on Through

All participants described a lifelong tendency to overlook feelings of discomfort. For Nina, “It's been a bit of a theme to my life that I tend to push on through”. Olivia normalised a life-long source of discomfort: “I’ve always had poor sleep”. Increased difficulties during perimenopause were dismissed as “another one of those poor sleeping things”. This capacity to be tolerant often harmed their wellbeing.

#### Accommodating Autism

Since menopause, Nina became aware that on a material level she was “needing recovery time a lot more”, although she suggested that “it's possible I just didn't realise I needed it”. Autistic identity enabled her to nurture her sensory needs—e.g., through music: “I almost feel my brain untangling”. Ella described more “frequent meltdowns” since menopause but also reported being able to recognise their warning signs more effectively.

You get an aura ... and you feel like ... there's a migraine coming on. And you can either carry on what you're doing or you can run away, go and lie in a darkened room ... and I'm beginning to get a sense of autistic situations being a bit like that y-you begin to feel like everything’s ... too much.

Her autism diagnosis gave her the legal entitlement and confidence to request accessibility modifications and felt less firmly obliged to “act normal” when overwhelmed: “It's not up to me to try and cover it up”. Similarly, Grace commented: “I’m brilliant at masking and actually, some of the time I really rather enjoy it… the question is whether I choose to… anymore”. She reflected on the sustainability of her pre-diagnosis persona: “What I thought was my best and most hilarious self was draining, basically pulling the plug out on my energy”. Recognition of the health benefits of unmasking lead her to question her identity: “Who the hell am I?”. Conversely, Olivia recalled:

When I accepted my self-diagnosis, which was just before I got my official diagnosis, my depression lifted greatly because understanding had come in, and a lot of stuff I was depressed about was to do with not understanding my place in the world.

Increased self-awareness was discursively attributed to surviving menopause itself, rather than to her autism diagnosis:

I think it's really important that I went through it as I have a greater sense of who I am. Had I skipped all of that by some, whatever, I would not be this self-aware, I'm pretty certain about that.

One consequence of self-awareness for her was choosing clothing that suited her sensory and temperature regulation needs. Nurturing her material body in this way was positioned as being conducive to a “reflective and contemplative” state. Engagement with strength training also helped her to embody a sense of being “strong, emotionally and physically” which allowed her to reject discourses of defeat and infirmity during this “time of struggle”. Newfound self-awareness enabled her to reduce her previous tendency to put others’ needs above her own: “I’m very good at problem solving but I was always using that skill to help other people… I was not able to do that for myself because I wasn't self-aware”. Menopausal identity was positioned by her as a cause for celebration: “Post-menopausal women are taking over the world, by the way!”.

#### Awareness of Age and Aging

Grace embodied conflicting discourses about ageing and self-awareness. She described “trying very hard to manage my energy in a different way” and had recently become aware of just “how terrible my self-awareness is”, a multi-layered intrapsychic process of becoming self-aware of her lack of self-awareness. For her, this shift was a necessity for managing increasingly challenging material circumstances linked to ageing: “I live with pain. It's tedious … I've been off work … my salary will be halved”. However, she described empowering aspects such as “being an older woman in public and having more authority” and subsequently reacting differently to injustice:

In the past I think that my body would react, and my body would go “Aargh!” but I would think ‘Don’t do anything. Don't do anything. Don't show any feeling’, whereas now, I don't necessarily show that I'm annoyed, but I will try and do something about it.

Additionally, she derived enjoyment from subverting younger people’s assumptions about older women by nonchalantly revealing her numerous piercings: “I think it's absolutely hilarious! ‘I'm not what you thought I was!’”. Her autism diagnosis enabled her to “feel OK to show that I was different”.

Initially, Ash embodied negative discourses about ageing: “Periods mean young and fertile and now I'm a little old crone”. However, Ash gradually subverted this positioning by accepting their “oncoming crone-ness”. By “figuring out the autism stuff, and the menopause”, their life-stage became “not really a mid-life crisis, more of a new wave”. Additionally, previously held priorities were put into perspective: “I gained weight around the middle” during menopause but “[I] don’t care about that kind of thing anymore”.

Ella positioned self-awareness and wellbeing as things that improve with age. She noted: “I've actually looked after myself better and have a better understanding of myself and have been happier and had better mental health as I've got older”, but she also acknowledged that autistic ageing “could be quite a scary prospect” for those who are not in a “privileged situation”, i.e., financial insecurity or limited social support. Lack of awareness, specifically about autistic ageing, was positioned as a concern: “We need to know what autism is like in the older person … especially if you have mental health difficulties” (Isla).

### Navigating Support Options

Most participants described conflicting or absent information about menopause management, particularly around the pros and cons of medical treatments. Peer support and NHS services were positioned as being helpful and effective by some participants, but inaccessible and frustrating by others.

#### Natural Versus Medical Management of Menopause

Biomedical approaches were positioned as being ominous and dehumanising by some participants. Dora explained that: “I wouldn't have had, say, a hysterectomy, unless it was all falling off and I was dying, so I have most of me intact and I want to stay that way”. However, she acknowledged the reasons why she and others would consider medical approaches: “When in the middle of something I am very much like, ‘This is terrible, you must surgically remove everything!’”. It is interesting to note the extreme terminology and images she deployed. Indeed, extreme disruption lead participants to reconsider their stance:

I was always one of those people who was like ‘No, I'm not going to take hormones, it's all a natural process’…and now I'm sitting here and going ... I haven't slept through the night in months” (Olivia).

However, some resisted an anti-medical discourse. For example, Ella advised that there is no reason to “put up with” discomfort. She positioned menopausal symptoms biomedically: as indicative of hormones that are “out of whack”, noting that Hormone Replacement Therapy (HRT) restores homeostasis and had helped her to accommodate her neurodiversity and manage her work life: “I'm still autistic … I'm still not like … superwoman, but … I'm much better able to do a sensible level”.

Nevertheless, the material discomfort of HRT side-effects was positioned by some participants as being as disruptive as menopausal symptoms themselves: “I woke up one day and said, ‘I can't live like this! it's making me brain dead!’” (Dora).

#### Interactions with Clinicians

Dora positioned the medicalised approach adopted by the NHS as inadequate, because “nobody was looking at the whole person and their experiences”. Temporary staffing meant that care “all got a bit separated” for Ella. After many years of having gynaecological issues dismissed, Nina received care from a specialist NHS service and recalled “feeling heard and listened to for the first time”. Isla described a positive working relationship with her GP who made autism-specific accommodations such as longer appointments and writing take-home notes because “he knows I can't retain the information”.

Grace, who has Ehlers Danlos Syndrome, a physical condition which frequently co-occurs with autism and increases the risk of organ prolapse (Eccles et al., 2012) was confused by contradictory advice from different doctors which complicated her treatment decisions:

I had the gynaecologist at [hospital 1] saying horrible things to me about, you know, ‘You must come off the HRT and blah, blah, blah’; And then when I went to [hospital 2] she said, ‘Why didn't they give you a hysterectomy? … She said, ‘It's keyhole surgery, it's not a big issue’. Then I was like, ‘Oh, OK, I don't know what to think’, because I've got, “It's not a big deal. It's just keyhole surgery’ ... and ‘You're going to get bladder and rectal prolapse’.

#### Subverting Taboos

Most participants alluded to the discursive positioning of menopause as a taboo topic. Dora described regularly challenging this societal custom:

I just say it in my everyday conversations, ‘Oh my God! It must be my hormones making me so hot’. They are like ‘OK!’. I think I have to be open about it because otherwise we are so embarrassed about it, it's just a fact of life.

She attributed her lifelong tendency to subvert taboos around gynaecological health to her neurodiversity: “I am now putting that down to being autistic really”. Additionally, she took it in her stride to challenge sexist assumptions held by doctors: “I refuse to be patronized by fully grown men who are overpaid… ‘Women of a certain age’ what does that even mean?”.

For more practical reasons, Ella subverted discourse of menopause as an “embarrassing” topic by explaining menopause to her teenage sons: “You’re having hormones too, right? … That's why I'm so … whatever … inept, incompetent, late, muddled”. Conversely, Grace positioned menopause secrecy as having an important survival function: “a flash of annoyance turns into a hot flush. So, the problem with that is it reveals feelings that you might be trying to conceal”. Thus, she was “very, very careful not to tell” not to reveal this to problematic men in her life. For her, menopause remained, “a sort of unspoken understanding between women of a certain age. If you suddenly get very hot you go like this, [mimes fanning]”.

#### Peer support

Peer discussions were positioned as a means of sharing practical advice: Ash was able to draw upon their lived experience of distress in order to offer support to a neurotypical friend who was struggling emotionally during menopause: “She's had good mental health so of course her menopause symptoms are a real bolt out of the blue for her. It was nice to be able to reassure her”. Peer conversations were especially valuable as “our mothers haven't told us anything … so we need to talk to each-other” (Ash). In contrast, Isla felt that this topic “does feel very personal”; she suggested that conversations about menopause between women have a bonding function which was not always desired: “I think she wants to get to know me better so she's talking about personal stuff but… she's not the right person to talk it over with”.

There were conflicting perspectives about the merits of peer discussions. For Ella, open discussions were valued, but she also described being mindful of others’ comfort and her neurodivergent tendency to overshare:

It’s maybe that autistic thing of - of … ‘Oh this thing is happening to me right now. I need to give you every single bit of information about it’. So, I'm trying to be a little bit wary of figuring out whether someone actually wants to talk.

Ella described social media as a uniquely suitable platform for autistic people to “join in and write a little or a lot depending on how they're feeling”. However, she cautioned that these settings could erupt into “arguments and infighting about small issues …Something that isn't core to supporting each other”. Social media were positioned as extremely affirming by Nina:

It's not just in my head and it's not just irrelevant. Sometimes I think I'm just making a fuss, but then when I to talk to someone else they are like ‘I have the same thing’.

This was especially helpful as she did not know many people in real life with similar experiences. Ash commented: “I’ve had more information off [social media] than my doctor”. Patient knowledge was positioned as more thorough than that of professionals: “I’ve found out myself and gone back to them with my advice” (Ash). For Isla, the “first point of call would be the internet … then my husband … then the doctor”. Social media were a useful platform for her to discuss physical health differences that other autistic people were also experiencing: “There’s another lady on [social media] with the same combination [of conditions].”. However, she felt “bereft” of evidence-based advice. Peer experiences and support were appreciated but this was partially in in lieu of relevant medical information which was “little and far between” (Olivia). As Nina put it:

If I'm unsure or worried about something I like to learn. For me, reading a book would be the biggest thing ... and then having the opportunity to reach out to other people.

Olivia identified societal reasons for an absence of information: “When it comes to medical research, women are so often ignored”, and noted that autism research is predominantly “directed towards children”; “It takes so long for it also to trickle down to professionals who seem to know even less than you know”.

## Discussion

This study was designed to examine how autistic people conceptualise menopause and to consider the implications of this for wellbeing, accessing support, and identity. Thematic decomposition revealed three discursive themes: *Uncertainty about Changes, Growing Self-Awareness and Self-Care,* and *Navigating Support Options.* These are discussed below in order to consider how the present study relates to and extends existing knowledge.

The theme “Uncertainty about Changes” referred to the difficulties identifying and understanding the physical and emotional alterations they were undergoing, particularly in the early stages of menopause. Autistic traits such as emotional and sensory sensitivity were heightened around menopause: menopause-specific changes such as hot flushes were described viscerally and were identified as being additionally uncomfortable due to their autistic sensory sensitivities. Limited understanding of menopause or indeed awareness of their autism was constructed as further complicating their experiences. These accounts are consistent with the experiences of autistic participants in Moseley et al.’s (2020b) study. However, in line with a minor finding of Moseley et al. (2020a), post-menopausal participants in the present study highlighted some positive changes: cessation of menstruation was considered a relief, and this is consistent with the finding that autistic people often have difficulties managing menstruation (Steward et al., 2018).

The theme “Growing Self-Awareness and Self-Care” covered participants’ accounts of events that occurred for them during midlife which were positioned as facilitating a shift in how they viewed themselves. All participants learnt of their autism in adulthood: for most, this provided a primarily positive and informative explanation for their lifelong differences. One participant did not consider her autistic identity to be especially significant, but she attributed an immense increase in self-awareness to her endurance and survival of a tumultuous menopause. Newfound self-awareness enabled interviewees to reconsider lifestyle- and appearance -related priorities, leading to the subversion of internalised cultural discourses around ageing and femininity. In the general population, positive constructions of menopause have been associated with positive perceptions of ageing such as increased wisdom and independence (Winterich & Umberson, 1999). In contrast, qualitative interviews with learning-disabled women revealed that menopause was experienced almost entirely negatively; favourable aspects of ageing such as independence were reported to be largely absent from their lives (McCarthy & Millard, 2003). Likewise, women with physical disabilities have described menopause as a “back-burner issue”, in relation to more pressing concerns (Harrison & Becker, 2007). Participants in the present study acknowledged that practicing self-care was important but was contingent upon favourable socioeconomic circumstances and the availability of appropriate advice and support.

The theme “Navigating Support Options” covered the various challenges that were encountered when attempting to access support. Although several participants described helpful interactions with clinicians, negative constructions were positioned as being linked to the absence of professional awareness of the healthcare and communication needs of autistic adults. Likewise, Moseley et al. (2020a, 2020b) reported that autistic people perceived a frustrating lack of professional support and knowledge and highlighted a need for better resources. In the general population, many women report feeling that their gynaecological complaints have not been taken seriously by practitioners (Tomlinson et al., 2017). Although not quoted in this paper, many of the participants described immense difficulties in trying to access an autism diagnosis. In particular, autistic people assigned female at birth may have to contend with doubt about the validity of their autistic identity as well as barriers to accessing support for their health and wellbeing concerns.

Most participants in the current study described engaging in a process of researching and constructing autistic menopausal wellbeing for themselves. Information from other autistic people on social media partially filled this support and knowledge gap. Shared experiences and understanding have been identified as an important feature of friendships between autistic people (Crane et al., 2020), and many women in the general population value peer discussions about menopause more than interactions with medical practitioners (Kenen et al., 2007). However, several participants in the present study highlighted that peer support was an inadequate substitute for evidence-based information and guidance. In terms of the *type* of information that is preferred, one study found that information about day-to-day autistic wellbeing was considered more important than information about causes (Pellicano et al., 2014). In the current study, biomedical approaches were constructed as being acceptable to participants on the premise that their ethos was “practical” rather than pathologizing and patriarchal.

Across themes, the topic of masking and camouflaging autistic traits was raised by participants, a proclivity that has been linked to poorer mental health (Cage & Troxell-Whitman, 2019). Moseley et al. (2020b) found that menopausal discomfort itself was presented as “cracking the mask of adaptive functioning”. Participants in the current study described lifelong difficulties recognising and describing internal bodily states, reflecting the common autistic trait of alexithymia (Poquérusse et al., 2018). This misrecognition led to an intrapsychic process involving self-doubt, frustration with their bodies, and attempts to ignore (or mask) their discomfort. Research has tended to define masking and camouflaging narrowly, solely in relation to social identity (Perry, 2019). However, participants constructed “unmasking” as encompassing internal processes in addition to social behaviours, such as using time alone to accommodate their sensory needs. Furthermore, although prior research has found alexithymia to be a relatively stable trait (Mikolajczak & Luminet, 2006), participants positioned self-awareness as something that could be cultivated over time: their masks were not cracked but purposefully chiselled off.

In the general population, women who are surrounded by medical discourse are more likely to report negative physical and psychological symptoms themselves (Ussher, 2008), suggesting that the biomedical discursive positioning of menopause may be harmful. However, when retrospectively describing menopause during their interviews, participants in the present study generally adopted a medical discourse, as illustrated by their identification of the role of “hormones”, and their reports that they were experiencing “symptoms”. This biomedical explanation was positioned as reassuring and contrasted to prior uncertainty. Although this could be attributed to an autistic tendency to dislike ambiguity (APA, 2013), a study of Japanese women found that despite menopause being positioned predominantly as a psycho-social rather than medical transition in Japan, factual information about bodily changes was empowering and helpful (Satoh & Ohashi, 2005). A study of learning-disabled women revealed that many experienced uncomfortable changes around menopause, but that until they were interviewed, they were unaware that menopause was an explanation for them (McCarthy & Millard, 2003). Similarly, limited prior awareness of menopause among autistic participants in the current study meant that these participants were somewhat shielded from apparently harmful medical discourse. However, a dearth of accessible medical information appeared to be problematic.

There is tentative evidence that autistic people can be especially sensitive to medication side-effects (Matson & Hess, 2011). There is presently no empirical research into the autistic experience of medical menopausal management. HRT was positioned as helpful for one participant but caused intense side-effects or additional complications for four other participants. Future research could utilise participatory methodologies to investigate the suitability of existing medical and non-medical treatment options and generate practical recommendations for healthcare providers.

This was the first study to investigate the autistic negotiation of menopause using a discursive approach which enabled nuanced exploration of participants’ positive and negative constructions. In order not to detract from the focus on language and participants’ own perspectives, relatively few demographic details were taken—e.g., ethnicity or psychometric scores. However, lacking this information may limit the generalisability of findings: experience of menopause appears to differ between ethnic groups (Sayakhot et al., 2012), but ethnic minorities are under-represented in autism research (Shaia et al., 2019). Furthermore, the online method of recruitment meant that respondents were likely to have been searching for information about menopause already, perhaps due to having particularly difficult menopausal experiences (Moseley et al., 2020b). Participants were extremely insightful and knowledgeable about autism: scientifically literate and articulate “expert patients” may not be representative of the autistic population as a whole (Department of Health, 1999) which may further limit the generalisability of the findings.

Although it is not possible to generalise to the diverse autistic population from this small sample of late-diagnosed autistic people, the analyses shed light on some of the ways that the autistic menopause is negotiated. However, the findings also highlight a need for more research into how best to support autistic people before and during menopause.

## Conclusions

Menopause was positioned by autistic participants as a complex transition time. Negative constructions were associated with uncertainty and difficulty identifying the causes of changes, which for most participants were physically and/or psychologically uncomfortable. Positive constructions were associated with increasing self-awareness as a result of survival of menopausal discomfort itself and late-diagnosed autistic identity. These informed subsequent coping strategies. Evidence-based, accessible information was highlighted as necessary, empowering, and helpful. Clinicians should ensure that they are aware of the communication and health needs of autistic people, and do not convey the implicit or explicit message that their experiences of menopause are “just in their heads” or “irrelevant”.
